# High Absorption of Electromagnetic Waves Based on 3D PMMA@Mxene@Co_3_O_4_ Composite Microsphere

**DOI:** 10.3390/ma17225427

**Published:** 2024-11-06

**Authors:** Jinghe Guo, Yanxiang Wang, Lanzhou Wang, Bohan Ding, Yongbo Wang, Yue Sun, Shichao Dai, Donglong Wang, Shishuai Bi

**Affiliations:** 1 Carbon Fiber Engineering Research Center, School of Materials Science and Engineering, Shandong University, Jinan 250061, China; 202334193@mail.sdu.edu.cn (J.G.); 202314123@mail.sdu.edu.cn (B.D.); 202314121@mail.sdu.edu.cn (S.D.); 2 School of Foreign Languages and Literature, Shandong University, Jinan 250100, China; 3 Shandong Jinhong New Material Co., Ltd., Weifang 262100, China; wdonglong2024@163.com (D.W.); jinhong098@163.com (S.B.)

**Keywords:** MXene, Co_3_O_4_, microwave absorption, composite materials, three-dimensional (3D) construction

## Abstract

With the increasing demand for effective electromagnetic wave (EMW) absorbers due to the proliferation of electronic devices and 5G communication systems, traditional wave-absorbing materials can no longer meet the current requirements. Thus, this research introduces a three-dimensional (3D) composite material consisting of PMMA@Mxene@Co₃O₄ microspheres, prepared through in situ self-assembly and hydrothermal growth. The strong electrical conductivity of Mxene, combined with the magnetic loss of Co₃O₄, ensures enhanced dielectric–magnetic synergy, leading to excellent EMW absorption. The study investigates the influence of varying Co₃O₄ content on the electromagnetic properties of the composite. Experimental results show that the optimal sample, with a thickness of 2.5 mm, achieves a minimum reflection loss (RL_min_) of −52.88 dB at 6.88 GHz and an effective absorption bandwidth (EAB) of 5.28 GHz. This work highlights the potential of 3D PMMA@Mxene@Co₃O₄ composites as high-performance microwave absorbers, providing a promising solution to EMW pollution. The findings offer valuable insights into material design strategies, demonstrate a promising pathway for developing lightweight, high-performance EMW absorbing materials by optimizing impedance matching and utilizing advanced microstructure design techniques.

## 1. Introduction

In recent years, due to the extensive development of electronic devices and 5G communication, the problem of electromagnetic wave (EMW) pollution has received more and more attention [1,2,3]. Although the traditional wave absorbers (such as carbon materials, ceramics, ferrite) in electromagnetic wave absorption have shown advantages, they are still unable meet the “wide, thin, light, strong” requirements [4]. The development of new wave-absorbing materials should not be delayed, as it is an effective way to solve EMW pollution [5,6]. Based on various reports, carbon nanotubes, graphene, and Mxene could signify very great developments in wave-absorbing materials; two-dimensional transition metal carbide/carbon nitride materials (MXene) due to their high electrical conductivity, possess a surface that contains a wealth of functional groups as well as a large surface area and other characteristics, widely used in electromagnetic wave absorption and shielding, and suitable for creating composite with magnetic substances. The combination of high dielectric and magnetic loss material achieves excellent microwave absorption (MA) performance through the optimization of material impedance matching and strong attenuation ability [7,8,9,10,11]. However, excellent impedance matching and high attenuation coefficients are usually constrained by each other, and the filling percentage of the absorber needs to be reasonably adjusted to obtain satisfactory experimental results of electromagnetic wave absorption strength, absorption frequency range, and coating thickness [12].

Mxene was first discovered by Gogotsi’s team in 2011 and was prepared by selectively etching the A component in the MAX phase. Mxene demonstrates great potential for electromagnetic wave absorption applications with the following characteristics: (1) with a monolayer or multilayer layered structure; (2) with a high dielectric loss; and (3) with a large number of surface defects and functional groups [13,14,15,16]. Several methods of synthesizing Mxene-based composites, with different structures and different physical phases to improve the microwave absorption properties, have been reported. For example, Deng et al. [17]. reported Mxene/Co_3_O_4_ composites with absorption better than 90% at a molar ratio of Ti_3_C_2_T_x_ to Co(NO_3_)_2_ of 1:9 and with a thickness of only 2 mm. However, the ultra-high dielectric value of Mxene leads to excessive reflection at the material interface and the lack of magnetic loss further hinders the application of Mxene-based MA materials. Therefore, Mxene-based composites such as Ti_3_C_2_T_x_/TiO_2_/Ni(NiO) and Mxene–rGO/CoNi have been developed as microwave-absorbing materials to further optimize the impedance matching. Gao et al. [18]. obtained three-dimensional floral Ni(NiO) on Ti_3_C_2_T_x_/TiO_2_, with an optimal RL at 14.96 GHz of −41.74 dB. Li et al. [19]. used vacuum-assisted filtration to prepare magnetized Mxene-rGO/CoNi thin films with an optimal RL of −54.1 dB at 13.28 GHz. At the same time, have been used there is a problem, in that the two-dimensional layered Mxene is prone to be re-stacked during processing, which leads to the limitation of microwave absorption performance.

Recently, researchers have been working to optimize impedance matching for microwave absorption properties through surface modification, composites of other materials, and effective design of microstructure and macrostructure. The above mentioned methods can be realized in the structural design of Mxene–magnetite composites. In situ self-assembly and template sacrifice strategies have been used to achieve the required reasonable structure, borrowing the magnetic loss of magnetic substances to match the high conductivity of Mxene to achieve impedance matching, and using advanced technology to simulate and test the various loss mechanisms to achieve the requirements of “wide, thin, lightweight, and strong” [20,21,22,23]. To meet the microwave absorption properties, three-dimensional Mxene composites were developed as wave absorbing materials, prepared by crosslinking, ultrasonic spraying, electrostatic spinning, rigid molding, freeze drying, and three-dimensional printing [24]. In addition to overcoming the restacked problem of 2D Mxene nanosheets, the three-dimensional structure it possesses can also increase the propagation path of electromagnetic waves for multiple reflections and absorptions, and the large specific surface area leads to intensive growth of magnetic substances, facilitating full use of the magnetic loss mechanism. In addition, the introduction of magnetic substances helps to harmonize the high conductivity of Mxene to achieve impedance matching, which greatly improves the performance of wave-absorption [25,26,27]. On this basis, the intercalation modification by acid or alkali can change the lattice spacing, the number of surface functional groups and groups of Mxene, introduce magnetic substances with different morphology and physicochemical properties, and reasonably assemble them to obtain excellent wave-absorbing agents through various preparation methods [28,29]. Co_3_O_4_, as a magnetic material, has been widely employed in a number of fields, including catalysts and magnetic materials. Researchers have devised a range of Mxene–magnetic composites for use in electromagnetic wave absorption, but bonding with Co_3_O_4_ has only been achieved at the two-dimensional level, and the corresponding loss mechanisms and preparation methods remain relatively underdeveloped.

Herein, a 3D PMMA@Mxene@Co_3_O_4_ composite microsphere sample is designed. PMMA is used as a template for the microspheres, in situ self-assembly is used to prepare the PMMA@Mxene composite microspheres, and they are combined by hydrogen bonding and van der Waals forces. The negative electronegativity groups on the surface of the Mxene act as a growth site for the Co^2+^, which is oxidized by a hydrothermal process into highly concentrated Co_3_O_4_ nanoparticles that are uniformly dispersed on the surface of PMMA@Mxene composite microspheres [30,31,32,33]. The advantages of this structural design are as follows: the strong conductivity of Mxene can obtain an excellent dielectric constant; a large number of defects exist at the interface; Co_3_O_4_ nanoparticles provide a guarantee for the magnetic loss of the system; and dielectric–magnetic synergy can be realized, which provides a reasonable design route and theoretical guidance for the preparation of high-performance electromagnetic absorbing materials. Compared with 2D Mxene@Co_3_O_4_, the self-stacking problem of Mxene nanosheets is effectively suppressed and the growth of Co_3_O_4_ particles is more homogeneous, which lays the foundation for good wave-absorbing performance; however, PMMA microspheres account for a larger proportion, and how to remove them becomes the only drawback. 

## 2. Experimental Section

The following materials were used: Ti_3_AlC_2_ MAX powder (≥98 wt% purity) from Jilin 11 Mxene Technology Co. (Jilin City, China) Lithium fluoride (LiF), concentrated hydrochloric acid (HCl), cobalt nitrate hexahydrate (Co(NO_3_)_2_·6H_2_O), urea (CO(NH_2_)_2_), and anhydrous ethanol (C_2_H_5_OH) were obtained from Shanghai McLean Biochemistry Technology Co. (Shanghai, China). Polymethyl methacrylate (PMMA) was purchased from Dongguan Dongcheng Kona Electronic Material Operation Department. All chemicals used without any pre-treatment.

### 2.1. Preparation of Few-Layered Ti_3_C_2_T_x_ Mxene Solution

According to a report, selective etching of the Al component in Ti_3_AlC_2_ MAX yields Mxene solutions. Ti_3_C_2_T_x_ Mxene solutions are obtained by etching a mixture of LiF and HCl in a certain ratio. Typically, 3.0 g of LiF was dissolved in 60 mL of 9 mol/L HCl solution, stirred for 30 min, and then 3.0 g of Ti_3_AlC_2_ was slowly added, and the reaction was carried out at 40 °C for 48 h. Subsequently, the black product was washed with deionized water and centrifuged several times until the pH value of the supernatant was greater than 6. After ultrasonication in an ice bath, the supernatant was centrifuged at 3500 rpm for 1 h, and the MXene solution was collected. The supernatant was collected as few-layered Mxene, with a Mxene concentration of 2.0 mg/mL.

### 2.2. Preparation of PMMA@Mxene Samples

200 mg of polymethyl methacrylate (PMMA) was dissolved in 20 mL of deionized water with micro-sonication and then dissolved with continuous stirring, and 25 mL of Ti_3_C_2_T_x_ Mxene solution was continuously dripped in with vigorous stirring for 4 h and left to stand for 30 min, then centrifuged at 5000 rpm for 10 min, and then centrifuged again after washing with deionized water. The PMMA@Mxene microspheres were then vacuum dried at 60 °C, after which they were named PM.

### 2.3. Preparation of 3D PMMA@Mxene@Co_3_O_4_ Composite Microsphere Samples

In situ growth of cobalt tetraoxide on PMMA@Mxene microspheres was achieved by hydrothermal reaction. An amount of 100 mg of PMMA@Mxene sample was dissolved in 60 mL of alcohol, and the ingredients were stirred to make the mixture homogeneous. Cobalt nitrate hexahydrate was used as the precursor for the preparation of tricobalt tetraoxide, and three different ratios (0.25, 0.5, and 1 mmol) of cobalt nitrate hexahydrate were added sequentially with vigorous stirring for 1 h. The solution was transferred into 100 mL of polytetrafluoroethylene inner liner, and was kept at 160 °C for 15 h, washed with deionized water and alcohol several times, and dried under vacuum at 60 °C for 6 h to obtain three-dimensional PMMA@Mxene@Co_3_O_4_ composite microsphere samples, which were named PMC-1, PMC-2, and PMC-3, respectively.

Characterization was conducted as follows: A powder X-ray diffractometer (XRD, Rigaku D/max-rC) with Cu Kα radiation was used for physical phase analysis at a scanning speed of 10°/min; an X-ray photoelectron spectrometer (XPS, Thermo SCIENTIFIC K-Alpha) was used to record the relative content of elements on the surface of the samples. The appearance and morphology of the samples were observed using a field emission scanning electron microscope (SEM, JSM-7610F), with 30 s of gold spraying to improve the clarity before the test, at a test voltage of 5 kv, and in conjunction with an energy spectrometer (EDS, JA5003N) for the analysis of the composition, at which time the test voltage was 15 kv. A high-resolution transmission electron microscope (HRTEM. JEOL JEM-2100) was used for further analysis of the microstructure of the samples, mainly observing the lattice fringes as well as the boundaries, ultrasonically dispersed in ethanol, at a test voltage of 200 kv. Electromagnetic parameters were measured by a vector network analyzer (VNA, Agilent Technologies N5244A) in the frequency range of 2–18 GHz. The measured samples were mixed according to a certain mass fraction (PMMA@Mxene@Co_3_O_4_:paraffin = 4:6) with paraffin wax, and pressed into a concentric ring specimen with an outer diameter of 7 mm and an inner diameter of 3.04 mm. The samples obtained in the experiment were all powder.

## 3. Results and Discussion

**Figure 1:**
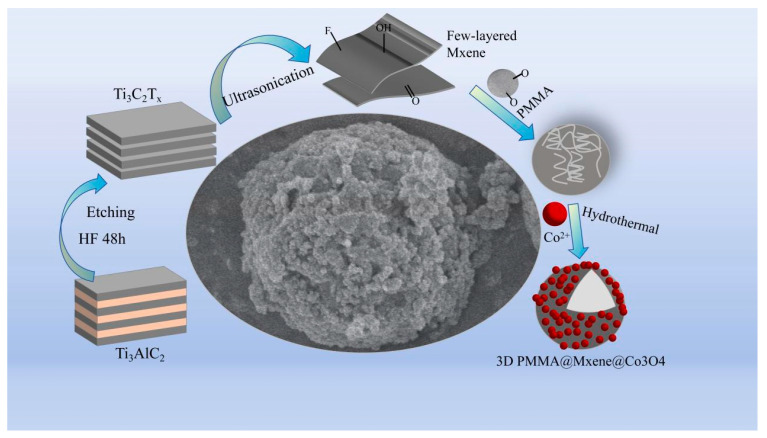
Schematic diagram of the preparation process of 3D PMMA@Mxene@Co_3_O_4_ composite microspheres.

Figure 1 illustrates the preparation process of 3D PMMA@Mxene@Co_3_O_4_ samples. Briefly, in situ self-assembly and hydrothermal growth methods were mainly used. Firstly, the A component in MAX was selectively etched with a mixture of LiF and HCl to obtain a multilayer Ti_3_C_2_T_x_ Mxene solution, which was subjected to ice bath sonication to disrupt the multilayer structure, and then centrifuged to collect the few-layered Mxene solution. The few-layered black Mxene solution could connect with the surface of white PMMA through hydrogen bonding and van der Waals forces to form a black precipitate with folds, which proves that the PMMA@Mxene (PM) microspheres were successfully prepared [34,35,36]. The etching process caused a large number of negatively charged groups (-F, -OH, =O) to be generated on the surface of Mxene, which provided the possibility of electrostatic adsorption growth of Co^2+^, which was oxidized to cobalt tetraoxide by a hydrothermal one-step method, and the 3D PMMA@Mxene@Co_3_O_4_ samples were finally obtained after vacuum drying by changing the content of cobalt nitrate hexahydrate [37,38].

**Figure 2:**
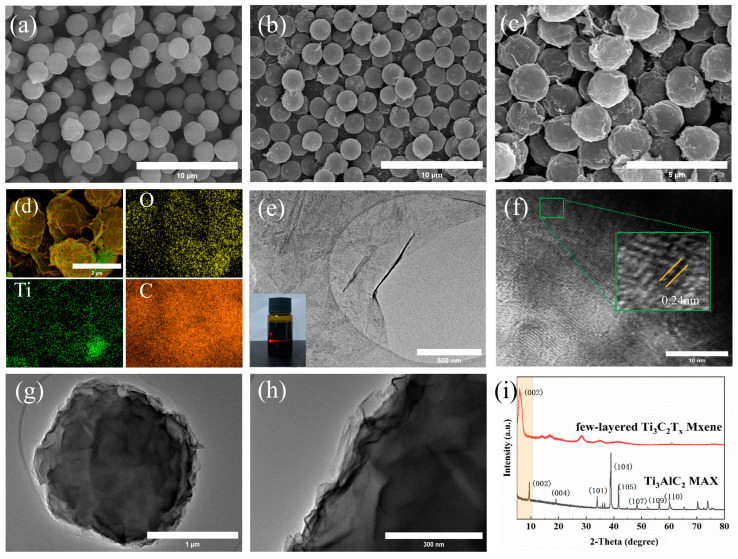
SEM images of the (**a**) PMMA; (**b**,**c**) PMMA@MXene; (**d**) elemental mapping images of PMMA@MXene, TEM and HRTEM images of the (**e**,**f**) few-layered MXene; (**g**,**h**) PMMA@MXene; and (**i**) XRD patterns of MAX and MXene.

The morphology and physical phase of the Mxene, PMMA, and PMMA@Mxene composite microsphere samples are shown in Figure 2. The SEM images of the PMMA samples are shown in Figure 2a. The microsphere surface is relatively smooth and uniform in size, with a diameter of 1.8 μm, and the few-layered Mxene nanosheets and PMMA microspheres are mixed together; the microsphere surfaces become rough, and the microspheres are connected to each other due to the bonding, with the surface grouping resulting in a small amount of agglomeration (Figure 2b,c) [39,40,41]. In addition, Figure 2d shows the results of the distribution of elements on the surface of the PM samples, with a uniform distribution of C, O, and Ti, thus confirming the success of PM capping. The TEM image of the few-layered Mxene sample is shown in Figure 2e, indicating the ultra-thin features after etching and stripping, with a thickness of only a few lamellae and the phenomenon of wrinkles, and the inset shows the Tyndall effect present in the Mxene solution. The crystalline spacing of 0.24 nm, which corresponds to (103) crystalline surfaces of the Mxene, as shown in the HRTEM image (Figure 2f). Figure 2g,h show the TEM images of PMMA@Mxene composite microspheres, which have lower contrast due to the lower molecular weight of PMMA, but it can still be clearly seen that the surface of PMMA microspheres is wrapped by several layers of Mxene and tightly stacked, and this is still regular spherical shape, due to the combination of electrostatic adsorption and hydrogen bonding; the surface of the microspheres is wrinkled, which means that the thickness of the Mxene lamellae cannot be determined. Compared with the MAX phase, Mxene exhibits (002) strong characteristic peaks, and the absence of (104) crystal facets near 39° further confirms the successful preparation of Mxene (Figure 2i).

**Figure 3:**
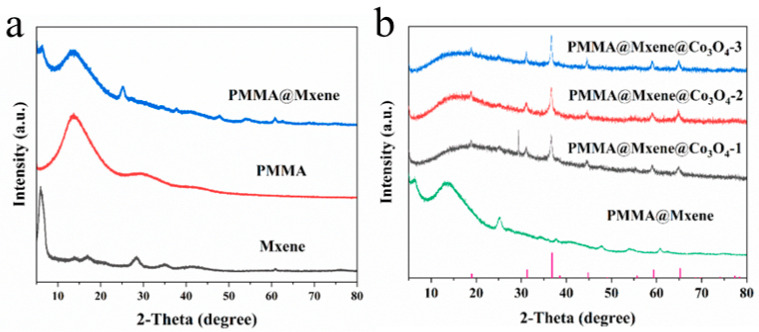
(**a**) XRD patterns of MXene, PMMA and PMMA@Mxene; (**b**) XRD patterns of 3D PMMA@Mxene@Co_3_O_4_ samples.

Figure 3a compares the XRD patterns of the Mxene, PMMA, and PMMA@Mxene samples; the broad peak located near 15° is characteristic of PMMA samples with poor crystallinity. The peaks at 2θ equal to 6°, 17.5°, and 61° correspond to the (002), (004), and (110) crystalline facets of Mxene [42], and the PM samples show a (002) crystalline facet compared to Mxene. The characteristic peak of the (002) crystal face is shifted to the right to 6.5°, which is caused by the absence of the surface group, and having both the 6.5° peak and the broad peak of PMMA located at 15° proves that the PM preparation is successful. The XRD patterns of 3D PMMA@Mxene@Co_3_O_4_ samples with different ratios are shown in Figure 3b, and the diffraction peaks at 19°, 31.3°, 36.8°, 44.8°, 59.4°, and 65.2° are the diffraction peaks at the cubic-phase tetrakisobalt tetraoxide (PDF#43-1003) (111), (220), (311), (400), (511), and (440) crystal planes [43], showing the high purity of Co_3_O_4_. The crystallinity of the Co_3_O_4_ samples increases with the increase of cobalt nitrate hexahydrate content, which is consistent with the XRD results. In addition, the (002) characteristic peak of Mxene is missing in the PMC sample because the dense Co_3_O_4_ on the surface blocks the X-ray signal.

**Figure 4:**
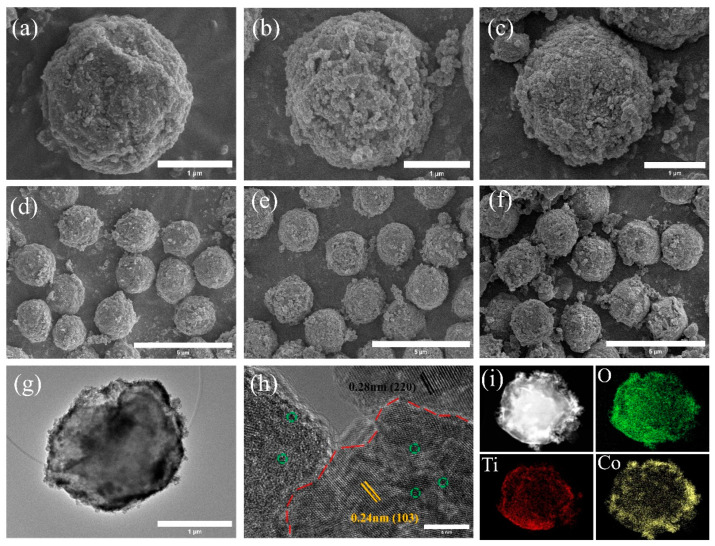
SEM images for 3D PMMA@Mxene@Co_3_O_4_ samples: (**a**,**d**) PMC-1; (**b**,**e**) PMC-2; (**c**,**f**) PMC-3, TEM and HRTEM images of the (**g**,**h**) PMC-2; and (**i**) elemental mapping images of PMC-2 sample.

After hydrothermal growth, the cobalt ions were oxidized into Co_3_O_4_ nanoparticles to obtain three-dimensional PMMA@Mxene@Co_3_O_4_ samples. The microscopic morphology of PMC-1, PMC-2, and PMC-3 samples is shown in Figure 4. With the appearance of PM microspheres having a similar spherical structure, the amount of Co_3_O_4_ on the surface of PM microspheres becomes more and more dense with the increase of cobalt nitrate hexahydrate dosage, and the nanoparticles have different particle sizes and different homogeneity. The scanned morphology of PMC-1 samples is shown in Figure 4a,d. The amount of Co_3_O_4_ on the surface is less, and the electronegative groups of Mxene, which are grown on the surface of the PM microspheres, are clearly seen, which is a demonstration that Mxene surface groups are deposition sites for Co^2+^ growth. When the content of cobalt nitrate hexahydrate is 0.5 mmol (Figure 4b,e), Co_3_O_4_ particles grow on the surface of PM sufficiently uniformly, a small amount of agglomeration phenomenon occurs, and a reasonable structural design is the best answer for obtaining excellent microwave absorption performance; when the content is further increased (Figure 4c,f), the aggregation of Co_3_O_4_ particles is serious, and agglomeration growth occurs, the surface homogeneity of PM microspheres is destroyed, and the inter-particle dispersion is poor. Meanwhile, the TEM images (Figure 4g–i) of the PMC-2 samples showed morphology and structure consistent with the SEM observations. The position of the black line in Figure 4h is the HRTEM image of PMMA@Mxene@Co_3_O_4_ samples, and it can be seen that the lattice spacing of 0.28 nm corresponds to the (220) crystalline plane of Co_3_O_4_ particles and the red line in the figure is the boundary between Mxene and Co_3_O_4_ particles, which is very clear; meanwhile, the defects are represented in the green circle, which provides a guarantee that excellent microwave absorption properties will be obtained [44]. Energy dispersive X-ray spectroscopy (EDS) mapping confirms the homogeneous dispersion of Co, O, Ti, and C elements on the PMC-2 spheres (Figure 4i).

When testing with a vector network analyzer, following three attenuation pathways typically occur between electromagnetic waves and the material: an impedance mismatch of the sample causes the electromagnetic wave to reflect off the surface of the sample; suitable impedance matching and losses (conductive losses, dielectric losses, magnetic losses, etc.) allow the electromagnetic wave to enter the interior of the sample for energy absorption; and certain electromagnetic waves are neither reflected nor absorbed by the sample, and thus pass through the sample and undergo transmittance. The microwave absorption capacity of an absorber is determined by the combination of the relative dielectric constant (ε_r_ = ε′ − jε″) and the relative permeability (μ_r_ = μ′ − jμ″) obtained from the measurement, which in turn calculates the wave absorption capacity. The real part (ε′) of the dielectric constant characterizes the size of the stored electrical energy of the sample, and the imaginary part (ε″) characterizes the size of the consumed electrical energy in the form of loss through conductivity, dielectric loss, etc. The real part (μ´) of the magnetic permeability indicates the absorption ability of the sample to the magnetic field, and the imaginary part (μ″) indicates the scattering ability to the magnetic field. The ratio of the imaginary part and the real part are used to evaluate the electrical loss (tanδ_ε_ = ε″/ε′) as well as the magnetic loss (tanδ_μ_ = μ″/μ′) capability of the material, respectively. The loss capability of electromagnetic waves is determined by the dielectric constant and magnetic permeability of the material itself and is frequency dependent, expressed by the attenuation coefficient α. The formula for calculating microwave absorption properties is as follows [45]:

Z = |Z_in_/Z_0_| = |(μ_r_/ε_r_)^1/2^tanh[j(2πfd/c)(μ_r_ε_r)_^1/2^]| (1)

RL(dB) = 20 lg|(Z_in_ − Z_0_)/(Z_in_ + Z_0_)| (2)

where Z_0_ is the impedance of the electromagnetic wave in free space, Z_in_ is the input impedance, ε_r_ is the relative complex permittivity, μ_r_ is the relative complex permeability, d is the thickness of the coating, f is the frequency of the electromagnetic wave, c is the propagation speed of the electromagnetic wave in free space (i.e., the speed of light), and j is an imaginary unit. Ideally, the closer the ratio of Z_in_/Z_0_ is to 1, i.e., the input impedance and free space impedance are perfectly matched, the better the incident electromagnetic wave can completely enter into the interior of the microwave absorbing material, and it can be efficiently consumed or converted into heat or other types of energy.

**Figure 5:**
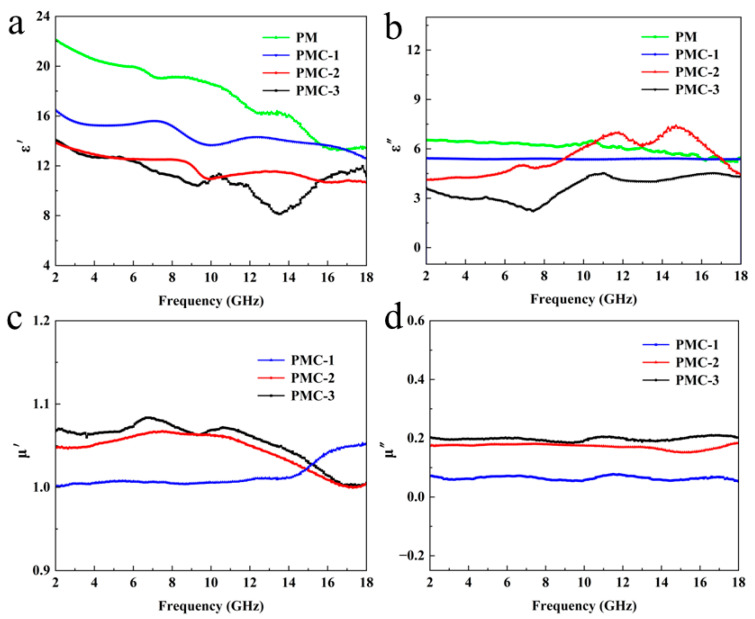
(**a**,**b**) Real and imaginary parts of permittivity of the PMMA@MXene, 3D PMMA@MXene@Co_3_O_4_. (**c**,**d**) Real and imaginary parts of permeability of the 3D PMMA@MXene@Co_3_O_4_ samples.

The electromagnetic wave absorption properties of PMMA@Mxene@Co_3_O_4_ samples with different precursor ratios were compared, and the electromagnetic parameters were measured in the frequency range of 2–18 GHz. The electromagnetic parameters include relative complex permittivity and relative complex permeability, which can be used to evaluate the electromagnetic properties of the samples. As expected, the PMMA@Mxene samples possessed higher dielectric constants, ε′ and ε″, with values in the range of 22–13 and 6.5–5.4, compared to the PMMA@Mxene@Co_3_O_4_ samples (Figure 5a,b), which can be attributed to the high electrical conductivity of Mxene, which allows a large amount of electromagnetic waves to be reflected at the surface instead of being absorbed into the interior. In addition, the polarization relaxation peaks in the figure are due to the interfacial polarization caused by the layered structure of Mxene; when the magnetic substance Co_3_O_4_ is introduced, the dielectric constant decreases, and the appropriate dielectric constant and structure match can make the material absorb and penetrate more electromagnetic waves internally. The values of the PMC-2 samples were in the range of 16.4–12.5 and 7.4–4.1. Two polarization peaks can also be observed in Figure 5b, indicating the presence of multiple polarization relaxation and conductivity loss, which is conducive to the improvement of electromagnetic wave absorption performance [46]. However, with the increase of frequency, the complex permittivity of the PMC-1 sample is approximately a straight line, and the conductivity loss generated by the current loop does not change much. Figure 5c,d show the magnetic permeability of the three samples, which is proportional to the content of cobalt nitrate hexahydrate precursor, and is omitted since PMMA@Mxene is not magnetic. Due to the lower Co_3_O_4_ loading on the PMC-1 sample, both μ′ and μ″ values are lower, and although the μ′ and μ″ values of PMC-2 are lower than those of PMC-3, due to the higher dielectric constant and thus increased dielectric loss, which improves the overall absorption performance, both μ′ and μ″ values are significantly higher than those of PMC-1 samples. Magnetic substances are magnetized in the external magnetic field, producing a hysteresis effect, eddy current effect, natural resonance, etc., which enhance the absorption of electromagnetic waves. Therefore, the appropriate dielectric constant and magnetic permeability play a very important role in the enhancement of electromagnetic wave absorption performance [47].

**Figure 6:**
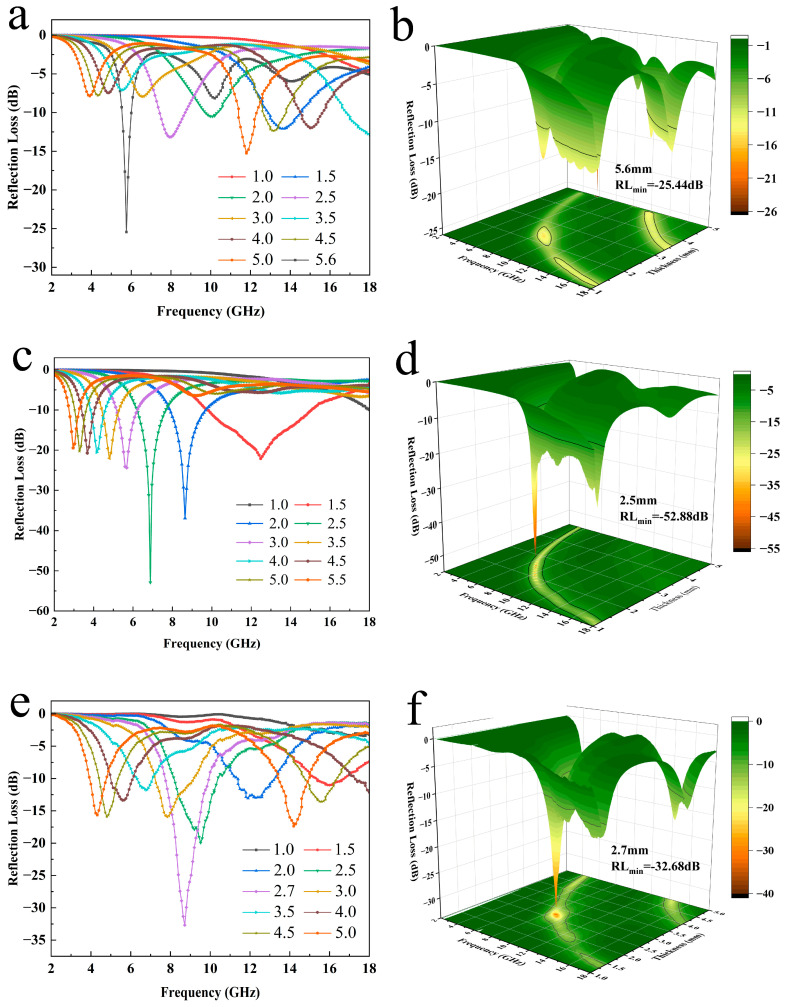
3D plots of the frequency dependence and RL curves of (**a**,**b**) PMMA@MXene@Co_3_O_4_−1; (**c**,**d**) PMMA@MXene@Co_3_O_4_−2; and (**e**,**f**) PMMA@MXene@Co_3_O_4_−3 samples.

Figure 6 shows the reflection loss (RL) values and the 3D plots of PMMA@Mxene@Co_3_O_4_ samples with different scales for thicknesses of 1–5 mm and frequencies of 2–18 GHz, respectively. According to the literature, based on the transmission line theory, the RL value is calculated from the complex permittivity and complex permeability. Theoretically, 90% of the incident wave can be absorbed when the RL value is less than −10 dB, and the corresponding frequency range is defined as the effective absorption bandwidth (EAB). All three allow 90% of the incident wave to be absorbed, as shown in the portion under the black line in the 3D diagram. As expected, the RL value of the PMC-1 sample is only −15.26 dB at 1–5 mm, which is slightly higher than the −18.28dB of the PMMA@Mxene samples, and reaches −25.44 dB only at 5.6 mm (Figure 6a,b), which is inevitably caused by the impedance mismatch. When the Co_3_O_4_ content is increased, the magnetic loss starts to play a role, the wave-absorbing performance of the composite microspheres is obviously strengthened, and the RLmin value of the PMC-2 sample reaches −52.88 dB at 6.88 GHz with an EAB of 5.28 GHz at a thickness of 2.5 mm (Figure 6c,d); it realizes the effective absorption in all frequency bands, with a strong absorption capacity and a wide EAB. The RLmin value of the PMC-3 sample reaches −32.68 dB at 8.72 GHz at a thickness of 2.7 mm, and thanks to the dielectric constant and magnetic permeability, its RL_min_ value is still lower than that of the PMC-1 sample. The frequencies corresponding to the minimum reflection loss values of the three samples are shifted to a higher frequency range as the sample thickness decreases, a phenomenon that can be explained by the quarter-wavelength (1/4λ) interference cancellation model:

t_m_ = nc/4f(|ε_r_||μ_r_|)^1/2^ (n = 1,3,5,7…) (3)

where t_m_ is the matching thickness corresponding to the lowest reflection loss. As mentioned above, the optimized ratio of 3D PMMA@Mxene@Co_3_O_4_-2 composite microspheres absorber has excellent electromagnetic wave absorption performance with the lowest reflection loss and the widest bandwidth, which proves that the combination of high-dielectric material and magnetic loss material obtains excellent microwave absorption (MA) performance through the optimization of the material impedance matching and strong attenuation ability.

**Figure 7:**
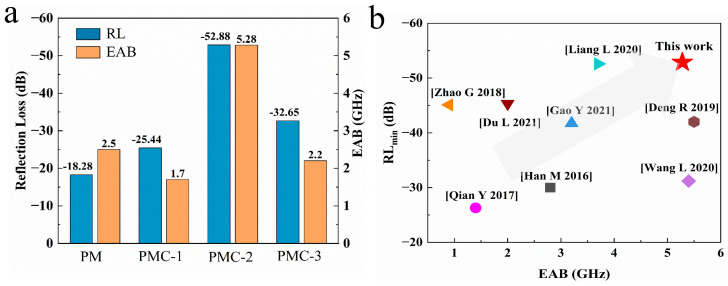
(**a**) Comparison of Rl_min_ and EAB of PMMA@Mxene and 3D PMMA@Mxene@Co_3_O_4_ samples; (**b**) RL and EAB of Mxene-based composites [3,7,18,19,25,34,43,44].

Figure 7a summarizes the RL_min_ values and the broadest EAB of PMMA@Mxene and 3D PMMA@Mxene@Co_3_O_4_ composite microspheres obtained at different ratios of cobalt tetraoxide for comparative analyses of MA properties. On the whole, cobalt nitrate hexahydrate level of 0.5 mmol is the best experimental condition for preparation. The best MA performance for PMC-2 is −52.88 dB for RL_min_ and 5.84 GHz for the broadest EAB. In comparison to PM microspheres, both the RL_min_ value and EAB of 3D PMMA@Mxene@Co_3_O_4_ are much smaller and broader. Figure 7b compares the RL values and the corresponding EAB of the Mxene-based composite microwave absorbers reported in previous studies, and the corresponding specific values are listed in Table 1. By contrast, 3D PMMA@Mxene@Co_3_O_4_ presents relatively high RL and broad EAB values, indicating that the construction of Mxene-magnetic has an important effect on the EMW absorption capacity.

**Figure 8:**
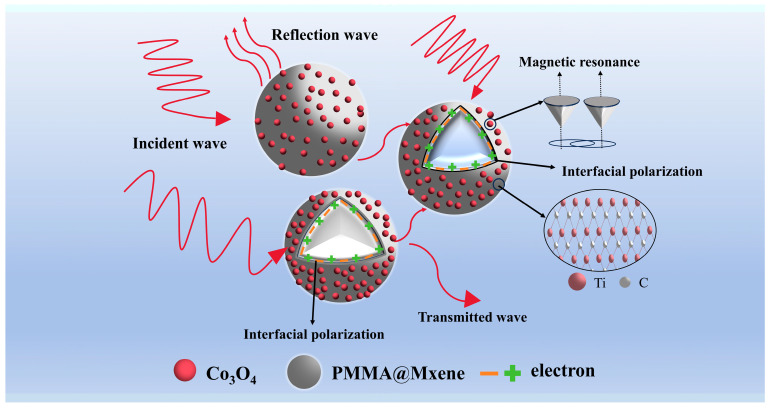
Schematic of electromagnetic wave absorption mechanism of 3D PMMA@Mxene@Co_3_O_4_ sample.

Based on the available characterization results and electromagnetic wave test data analysis, the 3D PMMA@Mxene@Co_3_O_4_ composite microspheres obtained by hydrothermal reaction have the best electromagnetic wave absorption performance when the precursor cobalt nitrate hexahydrate is 0.5 mmol (Figure 8). The reasonable pairing of the magnetic materials and Mxene helps optimize the impedance matching and ensures that the electromagnetic wave enters the interior of the material instead of being reflected at the surface. When the incident wave enters, the layered structure of Mxene forms a conductive network, which allows the incident wave to be transmitted and absorbed several times, as well as the migration change of electrons, which converts the energy of the electromagnetic wave into heat energy. At the same time, at the interface between Mxene and Co_3_O_4_, under the action of the external electric field, the ions or electrons in the dielectric collect at the interface, which can induce interfacial polarization, and the defects and vacancies can also induce dipole polarization. Finally, Co_3_O_4_ provides magnetic loss for the microspheres, and magnetic resonance and eddy current loss occur to consume electromagnetic waves.

## 4. Conclusions

In summary, 3D PMMA@Mxene@Co_3_O_4_ samples are prepared by an in situ self-assembly and hydrothermal growth method to investigate the key role in the microwave absorption field. By exploring the ratio of precursors to PMMA@Mxene microspheres and preparing the magnetic material Co_3_O_4_ by a one-step hydrothermal method, the Co_3_O_4_ nanoparticles are finally uniformly grown in situ on PMMA@Mxene microspheres. Due to the matched impedance, the collocation of various loss mechanisms, and the appropriate growth method of the structural components, when the thickness of PMC-2 sample is 2.5 mm and the filler content is 40%, the optimal RL_min_ value reaches −52.88 dB at 6.88 GHz, and the EAB is 5.28 GHz, which continues to meet the requirements of “wide, thin, light, and strong” for wave absorbers. This study demonstrates more convenient preparation of Co_3_O_4_ nanoparticles, where the combination of Mxene and Co_3_O_4_ is no longer limited to the two-dimensional level, and lays a good foundation for its modification treatment. The structure of Mxene-magnetic matrix composites is carefully designed to maximize the advantages of the dielectric and magnetic materials, which provides a new way of thinking about the structural design of the wave-absorbing agent. At the same time, the sample has limitations and its application in the military in today’s world is still a challenge; if the PMMA microspheres can be removed to prepare Mxene@Co_3_O_4_ hollow microspheres, this will greatly enhance their wave absorbing properties.

## Figures and Tables

**Table 1:** Comparison of EM wave absorbing properties of Ti3C2Tx MXenes-based composites.

Materials	Auther Year	EAB(GHz)	RLmin(dB)	Ref.
Ti_3_C_2_T_x_ MXene	Han, M 2016	2.8	−30	[3]
ZnO-Mxene	Qian, Y 2017	1.4	−26.3	[7]
Ni(NiO)/Ti_3_C_2_T_x_/TiO_2_	Gao, Y 2021	3.2	−41.74	[18]
CoNiO_2_/CoNi@Mxene	Du, L 2024	2	−45.33	[19]
MXene Ti_3_C_2_T_x_@RGO	Wang, L 2020	5.4	−31.2	[25]
Annealed Mxene/Fe_3_O_4_	Zhao, G 2018	0.9	−45.1	[34]
Ni@MXene	Liang, L 2020	3.7	−52.6	[43]
MXene/Co_3_O_4_	Deng, R 2019	5.5	−42	[44]
PMMA@Mxene@Co_3_O_4_		5.28	−52.88	This work

## Data Availability

The original contributions presented in the study are included in the article, further inquiries can be directed to the corresponding authors.
